# Existing Data Analysis in Pediatric Critical Care Research

**DOI:** 10.3389/fped.2014.00079

**Published:** 2014-07-29

**Authors:** Tellen D. Bennett, Michael C. Spaeder, Renée I. Matos, R. Scott Watson, Katri V. Typpo, Robinder G. Khemani, Sheri Crow, Brian D. Benneyworth, Ravi R. Thiagarajan, J. Michael Dean, Barry P. Markovitz, for the Pediatric Acute Lung Injury and Sepsis Investigators (PALISI)

**Affiliations:** ^1^Pediatric Critical Care, University of Colorado School of Medicine, Aurora, CO, USA; ^2^Critical Care Medicine, Children’s National Medical Center, Washington, DC, USA; ^3^Pediatric Critical Care Medicine, San Antonio Military Medical Center, United States Air Force, San Antonio, TX, USA; ^4^CRISMA Center and Pediatric Critical Care Medicine, University of Pittsburgh School of Medicine, Pittsburgh, PA, USA; ^5^Pediatric Critical Care, University of Arizona College of Medicine, Tucson, AZ, USA; ^6^Anesthesiology and Critical Care Medicine, Children’s Hospital Los Angeles, University of Southern California Keck School of Medicine, Los Angeles, CA, USA; ^7^Pediatric Critical Care Medicine, Mayo Clinic, Rochester, MN, USA; ^8^Pediatric Critical Care Medicine, Indiana University School of Medicine, Indianapolis, IN, USA; ^9^Pediatric Critical Care Medicine, Boston Children’s Hospital, Boston, MA, USA; ^10^Pediatric Critical Care, University of Utah School of Medicine, Salt Lake City, UT, USA

**Keywords:** pediatrics, intensive care, outcomes research, epidemiology, health services

## Abstract

Our objectives were to review and categorize the existing data sources that are important to pediatric critical care medicine (PCCM) investigators and the types of questions that have been or could be studied with each data source. We conducted a narrative review of the medical literature, categorized the data sources available to PCCM investigators, and created an online data source registry. We found that many data sources are available for research in PCCM. To date, PCCM investigators have most often relied on pediatric critical care registries and treatment- or disease-specific registries. The available data sources vary widely in the level of clinical detail and the types of questions they can reliably answer. Linkage of data sources can expand the types of questions that a data source can be used to study. Careful matching of the scientific question to the best available data source or linked data sources is necessary. In addition, rigorous application of the best available analysis techniques and reporting consistent with observational research standards will maximize the quality of research using existing data in PCCM.

## Introduction

“Big data” is the nickname in computer science, business, and public policy for the application of sophisticated analytic techniques to large and rapidly growing databases (1–3). Medicine has been lauded for its early adoption of data-driven “evidence-based” decision-making, but has been noted to be lagging behind other industries in leveraging the rich data available in electronic health records, registries, and enriched administrative databases (1, 3, 4).

Secondary use of existing data is an attractive option for disease epidemiology, quality and safety questions, health services research, economic analyses, comparative effectiveness research, and implementation and dissemination science. Existing data often describe “real-world” care and may be used to define current practice variation, to analyze natural experiments such as policy changes, and to estimate available sample sizes for prospective studies. Existing data may be used to conduct studies that are not amenable to a randomized trial format (5), for example in areas with limited equipoise: published guidelines with incomplete evidence, persistent variation, or controversy. These potential benefits are balanced by the data quality limitations of many existing data sources and the “numerous examples of poorly designed studies utilizing datasets ill equipped to answer the research questions posed of them” (5, 6).

Relatively few children require critical care (7). Overall, each pediatric intensive care unit (PICU) cares for a small number of heterogeneous patients with relatively rare diseases. Care has improved such that mortality is rare, but the risk of significant morbidity is high (7, 8). This distribution of patients and outcomes has made clinical research in pediatric critical care logistically challenging and expensive because appropriately precise estimates of effect require data from many centers (9). Despite these challenges, clinicians and researchers in pediatric critical care medicine (PCCM) have the potential to decrease a lifetime of disease burden for their patients.

Pediatric critical care medicine research differs from adult critical care research in that no dominant claims database analogous to Medicare exists; pediatric patients are usually reimbursed via a mixture of private payers and state-based Medicaid systems that are not uniformly reported. Large, multi-center existing data sources and linkage of multiple data sources may provide solutions to both challenges in PCCM research: the small sample size of any one patient type at each institution and the lack of a dominant claims database.

The objectives of this paper are to review and categorize the existing data sources that are important to PCCM investigators and the types of questions that have been or could be studied with each data source. Our goal is to provide PCCM investigators with resources to assist them in matching a research question with the most appropriate available data.

## Data Sources for Pediatric Critical Care Research

Choosing a data source for an analysis begins with carefully assessing the strengths and limitations of each data source. Investigators evaluating data source quality may benefit from using a tool that Black and Payne (10) developed and Cooke and Iwashyna (6) adapted for use with adult critical care data sources. That schema evaluates databases based on coverage (representativeness, completeness of recruitment, variables included, and amount of missing data) and accuracy (raw data collection, explicit variable definitions and rules, reliability of coding, independence of observations, and data validation).

Matching the level of clinical detail in the data source to the research question is also very important (Table 1). Evaluating causal relationships or conducting comparative effectiveness studies requires a high level of clinical detail to allow accurate adjustment for confounding by indication, severity of illness, and other factors (6). Identifying risk factors for an outcome may require only a moderate level of clinical detail, and descriptive epidemiologic studies or policy evaluations may require only a low level of clinical detail.

**Table 1:** **Level of clinical detail in existing data sources**.

Level of clinical detail	Data source contents
High	Many clinical variables such as vital signs, physiologic data, laboratory results, or severity of illness scores
Moderate	Some clinical variables or utilization data (medications, imaging studies, etc.) or charge/cost information
Low	Data limited to standard administrative fields such as demographics, length of stay, disposition, and diagnosis and procedure codes

The authors of this manuscript met in March, 2013 to discuss this topic. At that time, we developed a preliminary list of data sources considered important to PCCM that at least one author was familiar with. We also developed a preliminary method to categorize data sources. These were refined over the next several months. The data source types are arranged below from generally more clinical details to less, with example data sources for each type (Table 2). Examples of questions that have been answered or could be answered with each data source type are also provided. Because several of the authors are members or users of the data sources described, the initials of the primary authors for each section are noted.

**Table 2:** **Data source types in pediatric critical care research**.

Type of data	Clinical detail	Example data sources	Represented population	Accessibility/cost	Notes
Public use datasets from funded studies and networks	High	BioLINCC, CPCCRN, PECARN	Study-specific or network-specific	High/free	Often available online (e.g., www.pecarn.org and www.cpccrn.org), requires data use agreement and IRB approval. May have limited data support
Pediatric critical care registries	High	Virtual PICU systems (VPS)	119 participating hospitals (fee) with ~600,000 PICU cases	Moderate/free to VPS members	Use by non-members requires partnership with a member hospital investigator. Requires review by the Research Committee, which is primarily intended to ensure that multiple investigators are not attempting to answer the same question
	High	ANZPIC registry	24 PICUs in Australia and New Zealand	Moderate/free	
	High	PICANet registry	32 PICUs in the United Kingdom and Ireland	High/free	May be merged with continental European PICU registries in the future
Therapy-specific registries	High	Society of thoracic surgeons – STS congenital database	Children who have undergone cardiac surgery at participating centers	Moderate/low	Cardiac anesthesia-specific data was introduced in 2010. Linked to PHIS
	Moderate	ECMO registry of the Extracorporeal Life Support Organization	230 voluntarily contributing centers. Internationally representative sample of ECMO utilization	Moderate/free	Available to member centers, special requests may be made to the ECMO Registry steering committee. Minimal ability to risk adjust, but plans in place to improve this in 2013. Also contains neonatal and adult ECMO runs. Interpretation of outcome and complication data should be done with care
Disease-specific registries	High	Department of Defense Trauma Registry	U.S. military, coalition soldiers, and civilian trauma patients	Moderate/free	Department of Defense only. Use requires partnership with a military investigator
	Moderate	American Heart Association Get With the Guidelines – Resuscitation	>400 voluntarily contributing hospitals (hospitals pay a fee to participate as a quality improvement initiative)	Moderate/free	Use requires approval of research request by AHA-GWTG-R Research Task Force
	Moderate	National Trauma Data Bank	>700 voluntarily contributing hospitals and >100,000 pediatric trauma admissions each year	High/$300 per year	Obtained from the American College of Surgeons. A nationally representative sample of adults treated at Levels I and II facilities is also available for purchase
Population-based registries	High	Rochester Epidemiology Project (REP)	All residents of Olmsted County, MN from January 1, 1966 to the present, with ~500,000 individuals and ~1.2 million records	Moderate/free	Unique population-based resource. Use requires permission from the REP obtained through online application
Quality improvement or benchmarking databases	Moderate	Pediatric Health Information Systems (PHIS)	44 free-standing children’s hospitals, >7 million inpatient cases and 20 million Emergency Department encounters	High/free to member hospitals	No physiologic variables. Resource utilization and charge data are detailed, but results of tests and studies are not currently widely available. Several linkages completed or planned
Claims databases	Moderate	State medicaid files	Data available from 1999 to present for all 50 states and D.C.	High/~$1,000–1,500 per year, per state	Limited use to date in PCCM research
Government administrative databases	Low	Healthcare Cost and Utilization Project (HCUP) databases (details below)			Prices for HCUP products frequently discounted for students
		Kids inpatient database (KID)	Every 3 years: 1997, 2000, 2003, 2006, 2009	High/$200–350 per year	Allows national-level estimates of pediatric conditions. Sample weighting requires analytic adjustment. Two to three million hospital discharges in each file
		National Inpatient Sample (NIS)	Annual ~20% stratified sample of hospital discharges, ~1,000 hospitals per year	High/$160–350 per year	Sample weighting requires analytic adjustment
		National emergency department sample (NEDS)	Annual ~20% stratified sample of ED visits in 28 states, 2006–2010	High/$500 per year	Linked to state inpatient databases to determine ED outcomes. Sample weighting requires analytic adjustment
		State inpatient databases (SID)	All inpatient discharge abstracts in participating states	High/~$35–3,000 per year, per state	Component files of the NIS and KID
		State emergency department databases (SEDD) files	All ED visits that do not result in admission, for each participating state	High/~$35–3,200 per year, per state	Component files of the NEDS. Information about patients seen in an ED and admitted is found in the corresponding SID

*Level of clinical detail: high = includes many clinical variables such as vital signs, laboratory results, or severity of illness scores. Moderate = includes some clinical variables or utilization data (medications, imaging studies, etc.) or charge/cost information. Low = data limited to standard administrative content such as demographics, length of stay, disposition, and diagnosis and procedure codes*.

### Public use datasets from funded studies and networks (Tellen D. Bennett/J. Michael Dean)

#### Examples: public use datasets from the Collaborative Pediatric Critical Care Research Network (CPCCRN) and the Pediatric Emergency Care Applied Research Network (PECARN)

National Institutes of Health (NIH)-funded studies and networks are now required to produce and make available a public use dataset after study completion. The scope of these datasets may be narrow in keeping with the study population, but the level of clinical detail is often very high. Collaborative Pediatric Critical Care Research Network (CPCCRN) and Pediatric Emergency Care Applied Research Network (PECARN) each have several public use datasets. Datasets from individual funded studies and from other networks such as the Canadian Critical Care Trials Group may be available by directly contacting the primary investigators. Datasets that are known to be available include studies of prone positioning in acute lung injury (11), activated protein C (12), and restrictive transfusion thresholds (13). Farris et al., for example, recently published a study of functional outcomes in children with severe sepsis using a dataset from the activated protein C trial (14).

### Pediatric critical care registries: United States (Robinder G. Khemani/Barry P. Markovitz)

#### Example: Virtual PICU systems (VPS), LLC

Although the main aim of Virtual PICU systems (VPS) is to provide comparative data for benchmarking and quality improvement, it is structured as a PCCM registry. It includes nearly 120 pediatric and pediatric cardiac ICUs from 100 participating sites, including some outside North America. One particularly useful aspect of VPS is that it contains severity of illness scores including Pediatric Risk of Mortality (PRISM) III, Pediatric Index of Mortality (PIM) 2, Pediatric Logistic Organ Dysfunction (PELOD), and several cardiac intensive care unit complexity scores.

All institutions report a minimal dataset of required elements. Additional, non-mandatory data are available for a large proportion of cases; each institution decides whether to report each class of non-mandatory data. This allows inference about specific procedures or diagnoses at the patient level if analysis is performed accounting for the institutional profile. Investigators have used VPS to answer questions regarding quality improvement and severity of illness in PICUs (15–17), and to analyze risk factors for outcomes in several specific cohorts of patients (18–20).

### Pediatric critical care registries outside the United States (Michael C. Spaeder)

#### Examples: the Australia New Zealand Paediatric Intensive Care (ANZPIC) Registry and the Paediatric Intensive Care Audit Network (PICANet)

A number of national and multi-national pediatric critical care databases and registries are maintained worldwide. Similar to data sources in the United States like VPS, the primary purpose is benchmarking among institutions. Recently, these data sources have been increasingly used for clinical research. The Australia New Zealand Paediatric Intensive Care (ANZPIC) Registry includes data from 24 PICUs in Australia and New Zealand. Publications include investigations of outcomes related to acute lung injury (21) and hyperglycemia (22), as well as inter-unit practice variation in duration of respiratory support (23) and length of stay (24).

Similar in content to ANZPIC, Paediatric Intensive Care Audit Network (PICANet) maintains data from 32 PICUs in the United Kingdom and Ireland. Investigations employing PICANet include studies of physiologic associations in the post-cardiac arrest population (25), utilization of palliative care services following PICU discharge (26), acute disseminated encephalomyelitis (27), and diabetes (28). A collaboration to create a standardized European pediatric critical care dataset is currently underway between PICANet and the owners of databases in the Netherlands, Italy, and Portugal.

### Therapy-specific registries (Sheri Crow: STS-CHSD and Katri V. Typpo/Ravi R. Thiagarajan: ELSO)

#### Examples: the Society of Thoracic Surgeons Congenital Heart Surgery Database (STS-CHSD) and the Extracorporeal Membrane Oxygenation (ECMO) Registry of the Extracorporeal Life Support Organization (ELSO)

The Society of Thoracic Surgeons Congenital Heart Surgery Database (STS-CHSD) was developed in 1994 (29) and now contains data from 108 U.S. centers, representing 86% of the 125 U.S. pediatric cardiac surgical programs, and three of the eight centers in Canada (30) [personal communication to Sheri Crow from Jeff Jacobs, and Marshall Jacobs]. Participating centers submit data about congenital heart surgery procedures including patient risk factors, surgical complexity scoring, operative techniques, care processes, and clinical outcomes. The data are available to participating hospitals, physicians, and the healthcare industry for benchmarking, quality improvement, and research.

The STS-CHSD data specifications are upgraded every 3 years. The 2010 upgrade included new fields facilitating improved long-term outcome assessment and linkage to other databases for pediatric cardiology and critical care. The STS-CHSD now includes data from 36 centers regarding the anesthetic techniques (31) used for congenital cardiac surgical procedures. The STS-CHSD has been used to study delayed sternal closure (32), perioperative corticosteroid use (33), pediatric cardiac surgical case volume (34), and gender and race effects on surgical outcomes (35).

The Extracorporeal Membrane Oxygenation (ECMO) registry of Extracorporeal Life Support Organization (ELSO) captures information on ECMO use for any indication. The registry was started in 1984 but contains data from patients supported with ECMO since 1976. Approximately 230 U.S. and international ELSO members submit data. Data elements include diagnosis and procedural information, pre-ECMO level of illness and support, indication for ECMO [pulmonary, cardiac, or to support cardiopulmonary resuscitation (ECPR)], ECMO equipment used, ECMO flow, duration of ECMO, and adverse events during ECMO. Outcome information collected includes survival to discharge, discharge disposition, and reason for death for non-survivors.

Each submission pertains to an individual ECMO run, with multiple ECMO runs possible for a given patient. Each patient has a unique identifier that can support patient-level analyses of outcomes, although a variable for ECMO center is not available. The database is currently being redesigned to improve data quality and to expand severity of illness information (36). Examples of analyses of ELSO data include studies of the use of ECMO in children with respiratory failure (37, 38), the use of ECPR in children (39, 40), and the use of different pump technologies to support children on ECMO (41).

### Disease-specific registries (Renée I. Matos)

#### Examples: the Department of Defense Trauma Registry (DoDTR) and the American Heart Association Get With the Guidelines Resuscitation (AHA-GWTG-R) database

The Department of Defense Trauma Registry (DoDTR) (formerly the Joint Theater Trauma Registry) includes U.S. military, non-U.S. coalition soldiers, and local civilian trauma patients (42). Data contained in the registry includes resuscitation information, injuries, procedures, Abbreviated Injury Scores (AIS), complications, burn details, blood products and fluids administered, medications, laboratory results, and disposition. An overview of the DoDTR has been published, and the registry has expanded with modifications to data collection, uniform definitions, and improvements in standardization (43). Although quality improvement is the primary mission of the DoDTR, several pediatric studies have used this data source (44, 45). It is unique because it contains a higher incidence of penetrating, blast, and burn injuries than is seen in most U.S. civilian hospitals.

The Association Get With the Guidelines Resuscitation (AHA-GWTG-R) (formerly the National Registry of Cardiopulmonary Resuscitation) began in 1999 and is a prospective multi-center registry of consecutive patients with in-hospital cardiac arrests (46). The registry contains facility, patient demographics, pre-event, event, patient outcome (specifically, return of spontaneous circulation, neurologic outcome, and survival to hospital discharge), and quality improvement data. Limitations of the AHA-GWTG-R include the lack of physiologic variables, such as laboratory results or vital signs, and the convenience sampling frame. Despite its limitations, it has been the source for several important analyses of critically ill children. Examples of research using the AHA-GWTG-R database include studies of the effects of patient age (46), heart rhythm (47), and cardiopulmonary resuscitation duration (48) on outcome after cardiac arrest.

### Population-based registries (Sheri Crow)

#### Example: the Rochester Epidemiology Project (REP)

The Rochester Epidemiology Project (REP) (49) is a research infrastructure system supported by the NIH that collects, archives, links, and indexes the medical records of virtually all individuals who have resided in Olmsted County, MN since 1966 (50). The REP supports near complete assessment of health care utilization by a population of children, allowing estimation of the true incidence and prevalence of pediatric critical illness within a geographically defined area. Furthermore, medical record linkage for county residents throughout their lifetime facilitates long-term follow-up (51). A 2011 census identified 41,332 children <20 years of age who were current residents of Olmsted County (49).

The REP has been used for epidemiologic studies on a wide range of medical conditions. Most recently, REP data linkage with Olmsted County school records was utilized to identify a higher rate of attention deficit disorder and learning disability in children with two or more anesthetic exposures prior to age 4 (52).

### Quality improvement or benchmarking databases (Tellen D. Bennett)

#### Example: the Pediatric Health Information Systems (PHIS) database

The Pediatric Health Information Systems (PHIS) database was originally created by the Children’s Hospital Association (CHA), a business alliance of 44 free-standing children’s hospitals, as a collective purchasing database (53). PHIS is now more often used for quality improvement, benchmarking, and research purposes. Data are readily available since January 1, 2001 (for some hospitals, back to 1992). The resource utilization data are the most unique feature of PHIS; specific codes are used for each medication, imaging study, laboratory test, nursing or respiratory therapy, hospital room, or supplied material. Although identifiers are masked within the database, patients have one medical record number at a given hospital, making longitudinal studies of hospitalizations possible. The PHIS+ project at six member hospitals includes laboratory, microbiology, and imaging results data (53).

The PHIS database has most often been used to identify practice variation (54), to track trends in utilization (55), and to analyze readmissions (56). In addition, some comparative effectiveness studies have been reported (57). Other work relevant to PCCM includes analyses of cardiac surgery (33), complicated pneumonia (58), asthma (59), and traumatic brain injury (55).

### Claims databases (R. Scott Watson)

#### Example: state medicaid databases

Medicaid claims data have been used in policy-related research in neonatal critical care (60), but they have not been well studied in pediatric critical care. Major potential advantages of Medicaid data are that they include longitudinal data on large populations of children, including use of inpatient, outpatient, and long-term care services; prescription medications; and durable medical equipment. Thus, they can provide information about events preceding and following a hospitalization for critical illness, including information regarding geographic patterns of care and regionalization. The challenges to working with Medicaid data are related to differences in Medicaid eligibility for families between states and over time. In addition, covered services for which data are available vary by state.

Medicaid data have become easier to obtain and analyze. Person-level data are provided as part of the Medicaid Analytic eXtract (MAX) files (61). Applications to obtain data are submitted through the Research Data Assistance Center.

### Government administrative databases (Brian D. Benneyworth)

#### Example: Healthcare Cost and Utilization Project (HCUP) databases

The Agency for Healthcare Research and Quality (AHRQ) developed the Healthcare Cost and Utilization Project (HCUP) databases to provide a source of nationally representative inpatient discharges. The National Inpatient Sample (NIS) and the Kids’ Inpatient Database (KID) contain inpatient data. Both are derived from the individual states’ State Inpatient Database (SID) files. The Nationwide Emergency Department Sample (NEDS), a compilation of State Emergency Department (SED) files is also available (62).

Most pediatric studies have used the KID, which has been aggregated every 3 years from 1997 to 2009. The 2009 KID contains data on hospital discharges for patients ≤20 years old from 4,121 hospitals in 44 states. The KID stratifies hospitals by geographic region, hospital control, urban/rural location, teaching status, bed size, and hospital type to obtain a nationally representative sample. Within each hospital, routine normal newborn birth-related discharges are then sampled at 10% while all other pediatric hospitalizations are sampled at 80%. This allows for excellent statistical power to detect rare diseases.

Healthcare Cost and Utilization Project datasets have been used in pediatric populations to study central line infections (63), chronic mechanical ventilation (64), complex chronic conditions (65), sepsis (66), and trauma (67).

## Database Linkage

Record linkage techniques can be used to connect the records of the same patient in two or more different data sources. Linkage can enrich data sources by bringing in complementary and/or longitudinal patient variables from different phases of care and expand the array of questions a single data source might be used to study (68). Database linkage is generally accomplished by deterministic (“direct”) linkage when databases share a unique identifier or by probabilistic (“indirect”) linkage when they do not. The PHIS database has been a center of recent linkage activity, including links to the STS database (69), the Children’s Oncology Group database (70), and the electronic medical records of six children’s hospitals (53). Other linkages of the PHIS database, including a linkage of VPS and PHIS, are in progress or have been proposed (Matthew Hall, PhD, personal communication to Tellen D. Bennett).

## Data Source Registry

This manuscript was necessarily selective in choosing databases to discuss, and it is likely that we are unaware of other rich data sources. To facilitate efficient and productive use of existing data, we have created a registry (http://vpicu.info/pedal/), where known data sources, their contents, and their availability are listed. A form on the site can be used to enter information about other data sources.

## Conclusion

Many sources of existing data are available to PCCM investigators. We have categorized a number of frequently used data sources, identified research questions for which they may be appropriate, and created an online registry of data sources. Two recent manuscripts have achieved some of these goals for researchers studying adult patients (6, 71). The growth of quality improvement and safety research, improvements in personal computer and statistical package capability, and the increasing number of investigators with skills in data analysis have contributed to the growth of research using existing data (6). Efforts to improve the quality of studies using existing data have resulted in several reporting standards for observational research, including the Strengthening the Reporting of Observational Studies in Epidemiology (STROBE) guidelines (72).

Overall, PCCM would benefit from increased linkage and integration of data sources to improve their granularity and level of clinical detail. Patient identifiers that would facilitate database linkage could dramatically expand the range of questions that could be answered with existing data. The privacy risks inherent in such a strategy are substantial and may require novel technological solutions. Similar risks are involved with inclusion of provider identifiers in large databases, but that will be necessary to accurately analyze within- and between-hospital variation. More clinical detail including the contents of electronic medical records systems, severity of illness measures, and longitudinal follow-up variables would improve the ability of investigators to fully understand the health of the population and the long-term impact of a PICU stay. Individual databases might accelerate their improvement processes by developing strategies to incorporate user feedback.

Efficiency is a major potential benefit of existing data analysis; a multi-center dataset may contain enough patients of a particular type to perform an adequately powered study at much lower cost and in a shorter period of time than a prospective study (73). The NIH is likely to be increasingly attentive to return on their investment in the future, and a large prospective study may not be funded if the question can be adequately answered using existing data (74).

Few of the datasets used by PCCM investigators are “big data” compared to those used in computer science, business, and public policy. However, many of them are “quirky and messy” (e.g., informative missingness, dependent observations, lack of a unique identifier, and evolving data standards) in ways that provide challenges to their use (75). Expertize in the analysis of existing data will be beneficial to investigators using these sources. New data types such as genomic data and signal data [e.g., the adult patient-focused Multiparameter Intelligent Monitoring in Intensive Care II (MIMIC II) database] often are “big data” and will present new challenges. Collection and analysis of waveform data (heart rate, arterial blood pressure, end-tidal carbon dioxide, etc.) from the PICU patients at many centers should be a goal for the future.

In conclusion, many existing data sources are potentially useful for PCCM investigators, and analyses of existing data are likely to have a growing impact on the field of pediatric critical care. Careful matching of the scientific question and the best available data source or linked data sources is necessary. In addition, rigorous application of the best available analysis techniques and reporting consistent with observational research standards will maximize the impact of research using existing data in PCCM.

## Author Contributions

Tellen D. Bennett, Michael C. Spaeder, R. Scott Watson, Katri V. Typpo, Robinder G. Khemani, Sheri Crow, Brian D. Benneyworth, J. Michael Dean, and Barry P. Markovitz designed the study, each author drafted at least one section and contributed to the tables, Tellen D. Bennett wrote the first draft of the manuscript, and all authors contributed to its revision. All authors have seen and approved this final version of the manuscript and agree to be accountable for all aspects of the work.

## Conflict of Interest Statement

The authors declare that the research was conducted in the absence of any commercial or financial relationships that could be construed as a potential conflict of interest.

## Abbreviations

AHA-GWTG-R, American Heart Association Get With The Guidelines Resuscitation; ANZPIC, Australia New Zealand Paediatric Intensive Care; CPCCRN, Collaborative Pediatric Critical Care Research Network; DoDTR, Department of Defense Trauma Registry; ECMO, extracorporeal membrane oxygenation; ELSO, Extracorporeal Life Support Organization; HCUP, Healthcare Cost and Utilization Project; KID, Kids’ Inpatient Database; MAX, Medicaid Analytic eXtract; MIMIC, Multiparameter Intelligent Monitoring in Intensive Care; NEDS, Nationwide Emergency Department Sample; NIH, National Institutes of Health; NIS, National Inpatient Sample; PCCM, pediatric critical care medicine; PECARN, Pediatric Emergency Care Applied Research Network; PELOD, pediatric logistic organ dysfunction; PHIS, pediatric health information systems; PICANet, pediatric intensive care audit network; PICU, pediatric intensive care unit; PIM, pediatric index of mortality; PRISM, pediatric risk of mortality; REP, Rochester Epidemiology Project; SED, State Emergency Department; SID, State Inpatient Database; STROBE, Strengthening the Reporting of Observational Studies in Epidemiology; STS-CHSD, Society of Thoracic Surgeons Congenital Heart Surgery Database; VPS, Virtual PICU Systems, Inc.
